# Fitness-for-Duty Testing: A New Approach to Workplace Safety

**Published:** 1995

**Authors:** Marcelline Burns, Susanne Hiller-Sturmhöfel

**Affiliations:** Marcelline Burns, Ph.D., is a research scientist at the Southern California Research Institute in Los Angeles, California. Susanne Hiller-Sturmhöfel, Ph.D., is a science editor of Alcohol Health & Research World

**Keywords:** drug free workplace, identification and screening for AOD use, employee related issues, performance test, AOD impairment, occupational health and safety, validity research methods

Alcohol and other drugs (AOD’s) impair cognitive functions and motor skills (Burns and Moskowitz 1980; Miller and Dolan 1974; Parker et al. 1977). Over the past 25 years, the risks associated with AOD-induced impairment have been well documented, especially for driving (Attwood et al. 1980; Chiles and Jennings 1970; Moskowitz and Robinson 1988). More recently, it has become clear that AOD-related impairment, as well as impairment from other causes (e.g., fatigue), also adversely affects performance in the workplace (Martin et al. 1994). Productivity declines and safety hazards increase, not only for the impaired worker but also for those who share the work environment with the impaired worker.

During the 1980’s, increasing concern about AOD effects in the workplace led to legislative measures to promote drug-free workplaces (Macdonald and Wells 1994). The requirements of these measures have been met in many companies by implementing drug testing programs, usually in the form of screening for the presence of drugs in the urine. The programs include preemployment, for-cause, and random screening approaches.

Drug screening programs, however, are controversial. Critics of drug screens view the procedures involved in obtaining urine specimens as an invasion of the employee’s privacy. Additional criticisms are that the tests do not detect alcohol; detect only a limited number of other drugs; and detect the drugs only when they are taken within a certain time period before the test, which differs among drugs. Finally, a very serious criticism of the drug screening approach is that although it provides information about the presence of a drug or drug metabolite, it does not determine whether performance is impaired as a result of this drug use (Butler and Tranter 1994).

These limitations of drug screens have given rise to a different approach to promoting safe workplaces. Performance-based fitness-for-duty (FFD) testing focuses on detecting the effect (i.e., impaired performance) rather than on identifying the specific cause of impairment (e.g., AOD use, medications, or stress). This article briefly describes the FFD testing approach and presents a model for the validation of FFD tests.

## Designing FFD Tests

Safety-sensitive jobs exist in many different workplaces. For example, in the transportation sector, drivers and pilots must be attentive and able to respond quickly and correctly. In the manufacturing industry, the operation of potentially dangerous machines requires skill and attention. In the health care field, doctors and nurses must make appropriate and timely decisions and perform difficult procedures. Because of different job demands in different workplaces, a fundamental question to be addressed by designers and potential users of FFD tests is, What skill(s) should the test assess?

Because not all jobs require the same skills, the question of what a performance-based test should measure is both difficult and important. Clearly, a test that can evaluate performance for only a few kinds of jobs will not suffice. On the other hand, it is not feasible to design specific tests for every job category. Therefore, FFD tests must be designed in a way that allows meaningful assessment of performance in a variety of jobs. Another requirement is that the tests must be brief so that they do not keep employees away from their work duties for more than a few minutes.

Currently available FFD tests use brief tasks to assess cognitive, psychomotor, or physiological functions, all of which have been shown to be impaired by AOD use. For example, in a critical tracking task, the employee attempts to control the random movement of an arrow and to keep it within a target area on a computer screen. In divided attention tasks, the employee must respond to two tasks that simultaneously appear on a computer screen. Responses are made on computer-type keyboards, tracking control devices, or touch screens. Still other tests require the employee to look into a view port and visually follow the movement of a small light.

Whatever the approach of a specific test, it must meet several criteria, as follows (Butler and Tranter 1994):

- The test must be sensitive to small changes in performance that occur after consumption of commonly used levels of AOD’s or in the presence of other conditions (e.g., fatigue). For example, alcohol begins to impair performance with any measurable blood alcohol concentration (BAC). Therefore, to be useful in the work-place, a test must detect impairment by alcohol at BAC’s at least as low as 0.05 percent.
- The test must produce reliable measures; that is, the results must be reproducible from one time to the next. With the advent of computer-based FFD tests, which are remarkably stable over time, this requirement can be fulfilled without difficulty.
- The test should be simple to administer, thereby avoiding undue economic and time burdens on the workplace and minimizing expensive training of personnel. It also should be brief and use equipment that is readily available and reasonably priced.
- The test’s scoring method should adjust for the learning that occurs as employees perform the test repeatedly, which could mask AOD impairment. Typically, this potential problem is handled by establishing a baseline performance level when the employee enters the testing program. This baseline is adjusted regularly to compensate for continued improvement due to practice or learning. To assess performance on any given day, the employee’s score is compared with his or her adjusted baseline score.
- The test should measure skills that are job relevant and critical to safe performance in many workplaces.

## A Model for Validating FFD Tests

Although several FFD tests currently are available, not all have been shown to be valid and reliable measures of impairment in the workplace. In part, the lack of validation can be attributed to a failure of test users to define what an FFD test *should* measure and a failure of test designers to specify exactly what a particular test *does* measure.

A proposed model for the much-needed validation of FFD tests is based on the assumption that safety-sensitive tasks require complex, rather than simple, skills. The performance of complex tasks involves such cognitive functions as perception, attention, memory, and information processing (Broadbent 1971; Forgus 1966; Neisser 1966). Consequently, unimpaired cognitive functioning is essential to the safe performance of complex tasks. According to this model, a valid FFD test must be sensitive to impairment of cognitive functions.

Validation of FFD tests using this model requires as a first step the use of a “gold standard” drug with known effects on cognitive functions to assess whether the tests reliably detect impairment. Alcohol serves well as such a gold standard for several reasons: It is the most widely available intoxicant and is a major contributor to impaired work performance; its impairing effects on cognitive functioning are understood better than the effects of any other drug or risk factor (e.g., Jones 1974; Parker et al. 1977); and it can be administered safely to subjects in validation studies.

Researchers have examined alcohol’s effects on cognitive functioning in the context of assessing driving skills across a wide range of BAC’s (e.g., Laurell 1977; Moskowitz 1973; Moskowitz and Robinson 1988). Based on the large number of studies that show impairment of driving skills by alcohol, legislators have established a set of BAC limits for driving, ranging from low levels for young people (0.01 percent) and commercial drivers (0.04 percent) to as high as 0.10 percent for the general driving population. These limits also could provide guidance for establishing BAC limits for workplaces.

Because of these characteristics of alcohol, examining FFD tests using alcohol and alcohol-induced impairment as a gold standard can be a valuable first step in the validation process. Tests that fail to detect alcohol-induced impairment reliably are not viable candidates for general use in the workplace. Tests that are sensitive to alcohol, on the other hand, can be subjected to additional studies with other variables (e.g., other drugs) or under more stringent conditions. Ultimately, validation requires assessment of a test’s sensitivity to a broad range of risk factors.

## Summary

Because drug testing based on body fluids is controversial and is limited in detecting AOD-induced impairment, brief FFD tests are an attractive alternative for the workplace. These tests assess different aspects of the workers’ performance. Before these tests are used widely, however, it is imperative to establish their validity. It is reasonable to begin the validation process by using alcohol as the gold standard drug.
